# Nutritional Challenges and Treatment After Bariatric Surgery

**DOI:** 10.1146/annurev-nutr-061121-101547

**Published:** 2024-08-12

**Authors:** Violeta Moize, Blandine Laferrère, Sue Shapses

**Affiliations:** 1 Obesity Unit, Hospital Clinic Barcelona and Institut d’Investigacions Biomèdiques August Pi Sunyer (IDIBAPS), Barcelona, Spain; 2 Centro de Investigación Biomédica en Red de Diabetes y Enfermedades Metabólicas Asociadas (CIBERDEM), Madrid, Spain; 3 Nutrition and Obesity Research Center, Division of Endocrinology, Department of Medicine, Columbia University Irving Medical Center, New York, NY, USA; 4 Department of Nutritional Sciences and New Jersey Institute for Food, Nutrition, and Health, Rutgers University, New Brunswick, New Jersey, USA; 5 Department of Medicine, Rutgers Robert Wood Johnson Medical School, New Brunswick, New Jersey, USA

**Keywords:** bariatric surgery, gastrointestinal, nutritional deficiencies, obesity, nutrition therapy, weight loss

## Abstract

Bariatric surgery is an important weight loss tool in individuals with severe obesity. It is currently the most effective long-term weight loss treatment that lowers obesity-related comorbidities. It also has significant physiological and nutritional consequences that can result in gastrointestinal complications and micronutrient deficiencies. After gastric bypass, clinical events that negatively affect nutritional status include malabsorption, dumping syndrome, kidney stones, altered intestinal bile acid availability, bowel obstruction, ulcers, gastroesophageal reflux, and bacterial overgrowth. Risk factors for poor nutritional status and excessive loss of lean body mass and bone include reduced dietary quality and inadequate intake, altered nutrient absorption, and poor patient compliance with nutrient supplementation. There are unique concerns in adolescents, older individuals, and individuals who become pregnant postoperatively. With careful management, health-care professionals can assist with long-term weight loss success and minimize the risk of acute and long-term nutrition complications after bariatric surgery.

## 1. INTRODUCTION

Obesity is a chronic disease characterized by excessive adipose tissue storage. It is often complicated by many comorbidities, including but not limited to type 2 diabetes mellitus (T2DM), hypertension, hyperlipidemia, cardiovascular disease (CVD), cancer, chronic kidney disease, hypoventilation syndrome, debilitating osteoarthritis, nonalcoholic fatty liver disease or steatohepatitis, severe acid reflux, and obstructive sleep apnea (7). These comorbidities negatively affect an individual’s quality of life and increase health-care costs. Obesity occurs worldwide, with some lower-income countries experiencing the highest increases in recent years (102, 103, 152). No country has reported a decline in obesity rates among their population. It is estimated that by 2035, over 4 billion people worldwide will be classified as overweight and obese [body mass index (BMI) ≥ 25 kg/m^2^], compared to over 2.6 billion in 2020. This represents an increase in obesity from 38% to over 50% of the global population during this 15-year period (155). The prevalence of obesity alone (BMI ≥ 30 kg/m^2^) is expected to rise from 14% to 24% during the same period, affecting nearly 2 billion adults, children, and adolescents by 2035 (155). Obesity rates are greater in adult women than men and in individuals with low education and socioeconomically disadvantaged groups. The sharpest increase in obesity is anticipated among children and adolescents, particularly boys (7, 129, 155).

The exact cause of obesity is not fully understood; however, there appears to be a complex interplay of factors such as genetics, neurohormonal mechanisms, obesogenic medications, sociocultural practices and beliefs, environmental factors, life experiences, and psychological factors (7). Food consumption patterns, urban development, sedentary behavior, and other lifestyle habits also influence obesity rates (129). To date, the best noninvasive interventions are dietary management and behavioral changes. Comprehensive, multicomponent interventions with intensive behavioral counseling and pharmaceutical approaches can produce meaningful weight loss (8). The incretin mimetics (e.g., glucagon-like peptide-1 receptor agonists) have shown success with significant weight loss (60, 153). However, because of side effects and cost, compliance decreasing over time, and weight regain occurring after medication cessation, long-term weight loss success remains to be determined. Currently, the most effective long-term weight loss treatment for severe obesity is bariatric surgery (BS), which is associated with a total weight loss of 15–30%, mostly sustained over time (20, 75).

Several studies have demonstrated that BS is associated with a favorable impact on all-cause and cardiovascular mortality, incidence of first occurrence of fatal or nonfatal CVD events, prevention and remission of T2DM, and quality of life (2, 118, 127). Consideration of BS as a therapy for severe obesity should be based on the balance of its benefits versus complications. The current laparoscopic approach for all BS procedures has resulted in a marked decrease in complication rates (106). Potential nutritional and gastrointestinal complications after BS are influenced by presurgical and postsurgical factors, surgical technique, postoperative weight loss, and patient adherence to nutritional follow-up (161). Patient education about the need for lifetime medical follow-up is crucial to detect and address nutritional problems to prevent severe malnutrition or other medical complications.

## 2. BARIATRIC SURGERY AS A TREATMENT OF SEVERE OBESITY

Because of the health benefits of BS, the use of this therapeutic approach for severe and complex obesity has increased (6, 118). However, it is important to acknowledge that BS carries risks, particularly in terms of nutritional complications due to changes in food intake and anatomical alterations (75, 92).

To ensure the best outcomes and minimize surgical complications, specific criteria have been established for selecting suitable candidates for BS. Thorough preparation is necessary before undergoing the procedure, as it is a life-altering intervention for managing obesity. Potential candidates undergo a multidisciplinary evaluation, including medical, psychological, nutritional, and functional assessments, to determine their eligibility and safety for surgery. Additional medical evaluations may involve cardiac, respiratory, metabolic, gastrointestinal, and sleep apnea testing. It is recommended that patients engage in behavioral interventions prior to BS and maintain those behavioral changes after BS as well (75).

Obesity is a requirement to be eligible for BS and is defined by BMI as class 1 (30–<35 kg/m^2^), class 2 (>35–40 kg/m^2^), and class 3 or severe obesity (>40 kg/m^2^). It is important to note that BMI has limitations since it is not a perfect reflection of body fat, and the threshold of obesity may not be generalized to people in Asia (22, 121). BS is indicated in individuals aged 18 years old and above a BMI of 35 kg/m^2^, who have at least one obesity-related complication, or for patients with a BMI of 40 kg/m^2^ or higher, regardless of the presence of obesity-related complications (39). BS may also be beneficial for patients with a BMI between 30 and 34.9 kg/m^2^, especially in patients of Asian descent who have unsuccessfully tried nonsurgical weight loss methods and experience obesity-related complications, particularly T2DM. The indication for surgery in these patients depends on the specific obesity-related complications and the potential long-term benefits compared to existing medical interventions (13, 20). Indications of BS in adolescents with severe complicated obesity are comparable to those of adults. However, the type of BS procedure has unique considerations and should reflect current pediatric guidelines (106). Additional information about adolescents is given below.

The choice of surgery type should be made in collaboration with a multidisciplinary team and consider the patient’s medical condition, anticipated outcomes, and benefits versus risks. Special attention should be given to patients with uncontrolled diabetes, type 1 diabetes, and insulin-treated diabetes. Short- and long-term remission (or the absence thereof) probabilities should be defined, as well as diabetes management during the perioperative period. Smoking cessation before BS is mandatory and should be maintained lifelong to reduce the risks of postoperative complications. Overall, BS combined with behavioral interventions in individuals with severe obesity is currently the most effective option for long-term weight loss and the control of chronic conditions.

### 2.1. Bariatric Surgery Procedures

**Figure 1:**
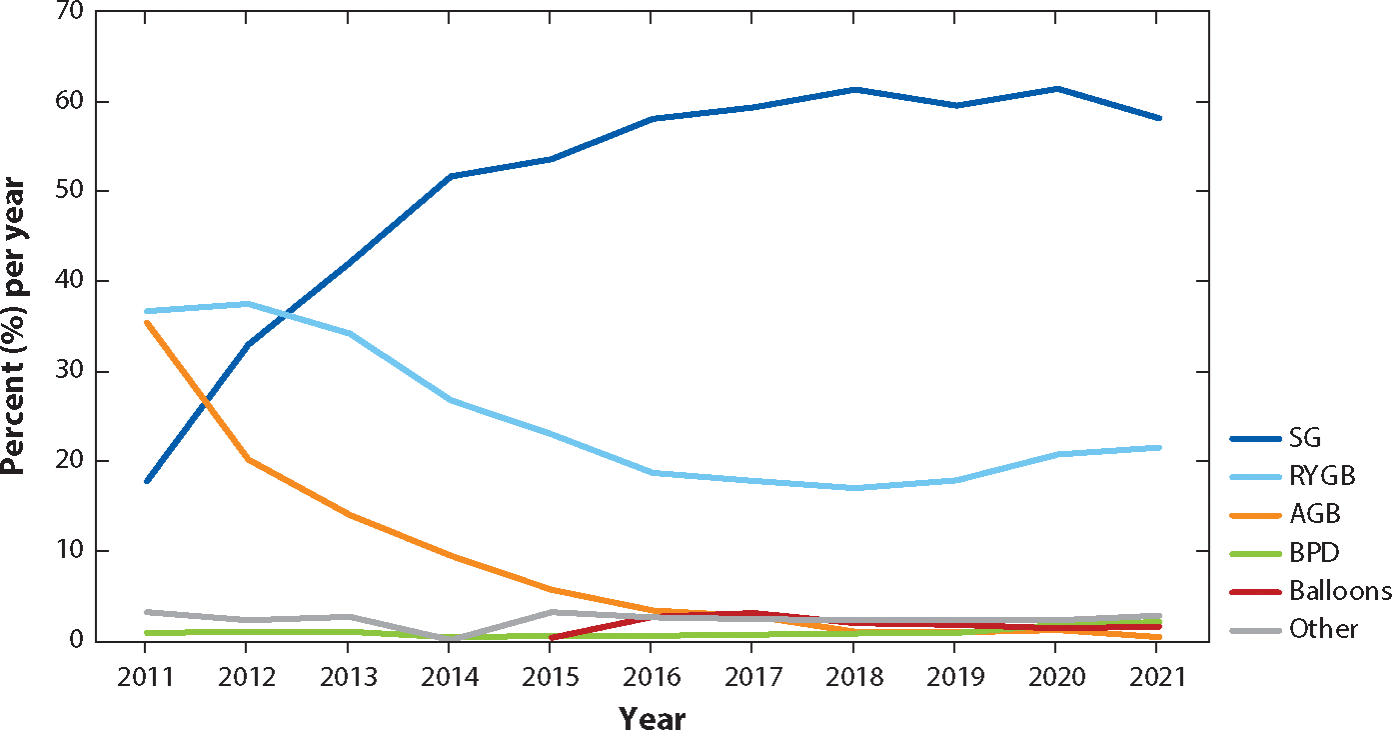
Bariatric surgical procedures (percent per year) for sleeve gastrectomy (SG), Roux-en-Y gastric bypass (RYGB), adjustable gastric band (AGB), biliopancreatic diversion (BPD), and endoscopic bariatric therapies (balloons and other procedures to reduce stomach size). Data from the American Society for Metabolic and Bariatric Surgery were used to create this figure (https://asmbs.org/resources/estimate-of-bariatric-surgery-numbers).

**Figure 2:**
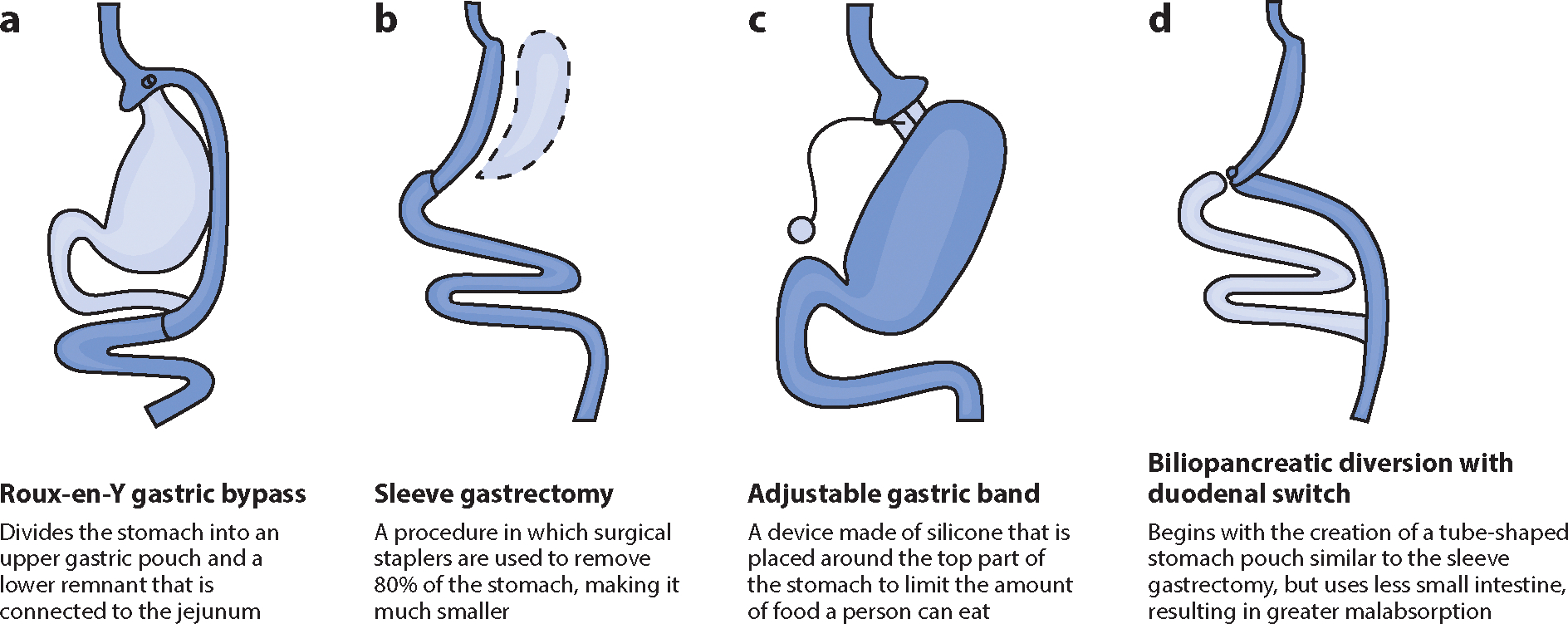
Bariatric surgeries: (*a*) Roux-en-Y gastric bypass, (*b*) sleeve gastrectomy, (*c*) adjustable gastric banding, and (*d*) biliopancreatic diversion with duodenal switch. Figure adapted with permission from Dr. Walter Pories.

Initially, BS techniques were categorized as restrictive, malabsorptive, or a combination of both, based on their presumed mechanisms for weight loss. However, these classifications have become outdated as it is now known that restriction and malabsorption may not be the only factors leading to significant and sustainable weight loss. Due to a better understanding of the metabolic changes resulting from different surgical interventions in the digestive system, BS is now commonly referred to as metabolic surgery. This term highlights that the surgical alteration of a normal organ modifies its function, partially influencing weight loss and other health benefits. For many years, Roux-en-Y gastric bypass (RYGB) was the most frequently performed BS procedure, followed by vertical sleeve gastrectomy (SG), adjustable gastric band (AGB), and, finally, biliopancreatic diversion (BPD), or its variant, duodenal switch (DS) (6) (Figure 1). SG has gained popularity and is currently the most performed surgical procedure. Mean postoperative weight loss at 1–2 years for RYGB, SG, and BPD ranges between 14% and 35%. Weight loss tends to be lower after AGB. The percentage of total weight loss decreases after 5 years to about 20% for RYGB and SG and 12% for AGB (9, 75). Current surgical techniques (Figure 2) are detailed below.

RYGB has been performed and studied extensively. It involves dividing the stomach into an upper gastric pouch (15–30 mL) and a lower gastric remnant. The gastric pouch is then connected to the jejunum, while the excluded biliary limb is reconnected to the bowel (88) (Figure 2a). In SG, the stomach is vertically transected, creating a high-pressure gastric tube and leaving a pouch of up to 200 mL. The pylorus is preserved, serving as the connection between the reduced stomach and the gut. Laparoscopic SG is now most commonly performed (Figure 2b). With AGB, a silastic band is placed around the stomach below the gastroesophageal junction and can be tightened or loosened using a subcutaneous access port and saline solution (Figure 2c). BPD and DS are procedures that involve a gastric component to reduce gastric volume and an intestinal component to decrease nutrient absorption. BPD includes a partial horizontal gastrectomy, while DS involves a vertical gastrectomy similar to SG. The small bowel is divided, and anastomoses are created to redirect the digestive tract (Figure 2d).

Single anastomosis duodenal-ileal bypass with sleeve (SADI-S) was introduced as a simplified alternative to BPD/DS (115) and has shown effectiveness in weight loss and resolving obesity-related comorbidities (114, 138, 143). It has also been successful as a primary procedure or revisional surgery (37, 116). As in all surgical procedures, there is potential for complications. However, side effects have been well tolerated and postoperative short- and long-term complications have appeared to be minimal (114, 123, 138).

Single-anastomosis gastric bypass (also known as omega loop gastric bypass/mini-gastric bypass/single-anastomosis bypass) is a procedure that has gained popularity, particularly in Europe and Asia (50). Several studies have demonstrated that it is a rapid, safe, and effective bariatric operation. It consists of a restrictive gastric pouch and a jejunal bypass with a single anastomosis, leading to significant fat malabsorption.

In addition to bariatric procedures, endoscopic bariatric therapies are nonsurgical procedures that are also used for weight loss. Endoscopic sleeve gastroplasty (ESG; 16–20% weight loss) and intragastric balloon (IGB; about 6% weight loss) are nonsurgical procedures that reduce stomach size, along with aspiration therapy. These therapies are typically not reimbursed by insurance and do not produce as much weight loss as bariatric surgical resections. They are performed in individuals with a lower BMI (30–35 or greater) who may not be eligible for other procedures or as part of research trials.

### 2.2. Surgical Complications/Adverse Events

Each surgical procedure has unique complications that can lead to nutritional deficiencies. Early complications of RYGB, SG, and BPD/DS include anastomotic leaks, small bowel obstruction, and dumping syndrome. The US National Patient-Centered Clinical Research Network consisting of 33,560 adults indicated that interventions, operations, and hospitalizations are relatively common after BS. Incidence increases after RYGB when compared to SG (29). Hernias, stomal stenosis, and staple line complications can occur weeks to months after the surgery. All of these complications typically require surgical intervention. Even though AGB is a less invasive procedure, adverse events such as band slippage, erosion, or prolapse require revisional surgery. The 30-day rates of major adverse events in a large study using the US National Patient-Centered Clinical Research Network were 5.0% for RYGB, 2.6% for SG, and 2.9% for AGB (9, 10). The AGB procedure necessitates band adjustment and often fails to induce weight loss in many patients, which may be a factor in the decline of AGB in recent years.

Adverse events from endoscopic procedures consist of mild to moderate symptoms such as abdominal pain and nausea (43). However, serious adverse events can occur. For example, complications from ESG include intraabdominal collection, hemorrhage requiring intervention, and refractory symptoms requiring reversal (135). There are fewer serious adverse events with IGBs than ESG, although there can be increased vomiting, nausea, and abdominal pain (110). The most common adverse events for aspiration therapy are postoperative abdominal pain, irritation at the tube site, peristomal granulation tissue, and nausea. These can generally be resolved with conservative management.

### 2.3. Obesity-Related Comorbidities

Surgical weight loss is accompanied by amelioration or resolution of most obesity-associated comorbidities such as dyslipidemia, hypertension, sleep apnea, fatty liver, and T2DM, regardless of surgery type. Improvement of these conditions is proportional to the magnitude of weight loss (16, 30). The procedures (RYGB, BPD, and SG) that show the greatest weight loss result in higher rates of remission of comorbidities.

The resolution of T2DM is perhaps the most spectacular one (17). Individuals with known short duration of T2DM (<2–4 years), with low glycosylated hemoglobin, that is controlled with few antidiabetic agents and who are not on insulin (i.e., who have substantial remaining beta cell function) are the most likely to experience diabetes remission. These patients typically undergo BPD, RYGB, or SG and lose a significant amount of weight. T2DM can go into remission for a few years in up to 80% of cases (25, 63, 83, 87, 128). The rate of remission varies greatly among publications due to vastly different cohorts in terms of T2DM duration and postsurgery date of assessment (128). Remission or improvement of T2DM for any duration is a relief for patients. As BS also improves dyslipidemia and blood pressure, overall CVD risk decreases substantially. Another benefit of surgical weight loss is the prevention of the development of T2DM in individuals with prediabetes (128).

The mechanisms of surgical weight loss and the resolution of complications, including diabetes, are not fully understood. The enhancement of the postprandial release of satiety gut peptides (84) and the change in reward brain control (132) may contribute to the larger weight loss after RYGB, BPD, and SG procedures when compared to AGB. In addition, the enhancement of incretins contributes to the improvement of beta cell function (48). Other mechanisms involving the microbiome (35), bile acids (4), decreased liver fat, decreased visceral fat (57), or a change in lipid absorption (62) could contribute to metabolic improvement and decreased cardiovascular risk (67).

## 3. NUTRITIONAL CONSIDERATIONS ASSOCIATED WITH BARIATRIC SURGERY

The risk of the development of nutritional deficiencies is influenced by various factors before, during, and after BS. Pre-existing impaired nutritional status is associated with postoperative nutritional deficiencies and metabolic complications (11, 34). Therefore, identifying and correcting nutritional deficiencies prior to surgery should be part of the comprehensive preoperative intervention (75, 79, 92). BS results in significant changes to gastrointestinal anatomy, which affects gut physiology and nutrient intake. Regardless of the surgical procedure, all patients experience changes in eating behaviors, including reduced portion sizes, and taste preferences. The potential for diarrhea and persistent vomiting contributes to reduced energy intake and limited intake of essential vitamins, minerals, and proteins. The combination of these factors results in a nutrient deficit (68, 73). The extent of nutrient malabsorption depends on the specific surgical technique, including the size of the stomach pouch, bypassing of the stomach, alterations in small intestine anatomy, and the length of the common channel (28, 92).

Along with these changes, reduced acid secretion due to gastric resection further impairs nutrient extraction and absorption modifications (28). Although the degree of impact on nutrition absorption may vary among different surgical procedures, all of them have been shown to negatively impact serum levels of iron, vitamin B_1_ (thiamin), folate, vitamin B_12_, and vitamin D, increasing deficiency risk (92). Studies on nutritional deficiencies have guided appropriate nutritional management (75, 92, 93, 99). It is generally understood that bypassing a longer segment of the intestine increases the risk of developing nutritional deficiencies. This makes resolving deficiencies more challenging, particularly in the long-term (53, 92). Importantly, adherence to nutritional supplementation (131) and regular medical follow-up after BS (19, 75, 100) play crucial roles in managing nutritional health.

Gastrointestinal symptoms such as dumping syndrome and gastroesophageal reflux can also have nutritional implications and contribute to changes in the gastrointestinal system (141). While data are available on the nutritional impact of popular procedures such as SG, RYGB, and BPD/DS, further research is needed to better understand the nutritional side effects of other surgical weight loss procedures and weight loss medications (92, 98).

Sustained negative energy balance is needed to promote weight loss. Dietary changes after BS entail calorie restriction. The protein content of the diet plays a crucial role during surgical weight loss. Adequate protein intake has been associated with the retention of lean body mass (LBM) (1, 78), satiety (84), thermogenesis (42), glucose homeostasis (specifically circulating levels of branched-chain amino acids after BS) (89), and the prevention of malnutrition (15). Due to the exclusion of the duodenum and proximal jejunum, where protein absorption primarily occurs, protein status can be significantly affected by BS (73, 75, 99). Furthermore, anatomical changes in the gastric pouch, including altered gastric acid secretion and pepsin production, affect the optimal digestion and absorption of dietary protein (15). It is widely acknowledged that not only the total amount of protein but also the presence of all essential amino acids in the diet are necessary for optimal protein synthesis and balance (105).

Malabsorption following BS can lead to increased nitrogen losses in feces, which may reach as high as 3.5 g/day compared to the normal estimated nitrogen loss of only 0.4 g/day. Increased nitrogen losses in feces may result in a negative nitrogen balance, where the body mobilizes protein stores from visceral tissues, impacting protein turnover. The decrease in LBM during weight loss can downregulate metabolic processes such as protein turnover and the basal metabolic rate, which may compromise long-term weight management (109). Conversely, a long-term negative nitrogen balance can lead to the loss of lean tissue, particularly affecting the adult population prone to developing sarcopenia, which is further discussed below.

## 4. GASTROINTESTINAL TRACT CHANGES AND NUTRITIONAL COMPLICATIONS

### 4.1. Malabsorption and Nutrient Deficiencies

Because RYGB, SG, and BPD/DS procedures alter the anatomy of the normal gastrointestinal tract and the ability to absorb nutrients, they raise postoperative nutritional risk. Despite high energy intakes, many people with obesity have micronutrient deficiencies even before surgery due to low-nutrient-density diets (49). Therefore, it is imperative that patients adhere to their individualized nutritional supplementation prescribed by their health-care team. There is a long history of vitamin and mineral deficiencies in postoperative BS patients (14, 16). The most common deficiencies are iron, vitamin B_12_, folate, thiamin, vitamin D, and calcium (Ca) (11, 49).

In patients with lipid or fat malabsorption, fat-soluble vitamin deficiency ensues. In contrast to BPD/DS, both RYGB and SG result in less fat malabsorption and very little carbohydrate malabsorption (91). For most micronutrients, studies have found similar micronutrient deficiencies present after both the RYGB and SG procedures, which is plausible because both procedures have similar malabsorptive aspects (66, 77). These studies and others found that the most common deficiencies in this patient population include vitamin D, iron, and vitamins B_6_, B_12_, and B_1_. However, one study indicated that patients with SG had higher hemoglobin (but not other iron markers), magnesium, and zinc compared to RYGB (and the AGB procedure) but lower folate levels (140). However, others have reported lower iron and vitamin B_12_ after SG (58). The pathological conditions associated with micronutrient deficiencies after BS are discussed below and shown in Table 1.

### 4.2. Vitamin B_1_, or Thiamin, Deficiency

The most common water-soluble vitamin deficiency in the BS population is B_1_, which is usually caused by low dietary vitamin B_1_ intake and hyperemesis. If left untreated, vitamin B_1_ deficiency can result in wet, dry, or cerebral beriberi. Wet beriberi is characterized by cardiac symptoms, including hypertension and tachycardia, while dry and cerebral beriberi result in neurological outcomes ranging from mental confusion to psychosis. Beriberi tends to develop 1–3 months after BS and has been reported to occur more often in females than males (136). It is possible to correct pre-existing deficiencies and prevent further deficiencies of thiamin with pre- and postoperative care.

### 4.3. Anemia

Anemia is reported after BS using malabsorption procedures. It is likely related to the type of surgery and the duration reported after surgery (134) but may be related to the presence of iron deficiency prior to surgery (55). Anemia can be secondary to high use of a postoperative proton pump inhibitor (PPI) in patients. PPI usage can lead to a reduction in hydrochloric acid production, which prevents the conversion of iron to the absorbable ferrous form as described in patients with laparoscopic SG (58). Iron deficiency anemia is a frequent problem in premenopausal women, often requiring parenteral iron therapy (52, 148). While iron deficiency is a major cause for anemia after BS, deficiency of vitamin B_12_ and folate, as well as zinc, copper, and selenium deficiencies, can also contribute (151). However, it is possible that many of these nutrient deficiencies are present before surgery because most studies do not examine preoperative to postoperative changes to specifically link the onset of anemia to BS.

### 4.4. Dumping Syndrome

A common intestinal complication after BS is dumping syndrome (101), which can occur following RYGB or SG (18). Dumping syndrome is the rapid passage of gastric contents into the small intestine, which commonly occurs with the consumption of simple carbohydrates, and can cause postprandial hypoglycemia or gastrointestinal disturbances. Treatment of dumping syndrome–induced hypoglycemia includes dietary counseling to decrease rapid carbohydrate intake and increase fibers and proteins in meals and snacks and the use of acarbose to slow glucose absorption (146), but there is no currently approved medication. After surgery, patients should be monitored for symptoms of dumping syndrome to prevent hypoglycemia and minimize gastrointestinal disturbances. Severe postprandial hypoglycemia can also occur and may be debilitating (112).

### 4.5. Bowel Obstruction

A possible risk factor with any abdominal surgery procedure, including bariatric procedures, is bowel obstruction. The etiologies range from blood clots to intestinal adhesions (41). Patients often have acute obstructions with symptoms of abdominal pain. Obstructions can occur after all types of BS, and therefore symptoms should be monitored since many require surgical intervention (95).

### 4.6. Ulcers and Gastroesophageal Reflux

Ulcers are one of the most common complications following bariatric procedures, especially RYGB, and typically present with gastrointestinal bleeding and severe abdominal pain (74). One study found that ulcers occurred in 3.5% of the population at an average of 7 months after surgery. All were successfully treated with PPIs and sucralfate therapy (32). Patient history of diabetes or peptic ulcers is a risk factor for marginal ulcers (139). While patients with BS are typically prescribed PPIs proactively, some patients still require surgical or other pharmacological interventions (81). Patients should be advised to avoid smoking and the use of nonsteroidal anti-inflammatory drugs, which increase the risk of ulceration (36). Ulcers that occur after BS can be treated proactively with pharmacotherapy and practicing general healthy habits to reduce the number of patients requiring additional surgical interventions. Gastroesophageal reflux disease (GERD) may improve in patients with obesity who undergo BS and weight loss, yet long-term follow-up suggests that there is a risk for GERD and Barrett’s esophagus after SG. The postoperative incidence of GERD, esophagitis, and Barrett’s esophagus can be up to 60% (69), with significant variability among groups (113). GERD after SG can be treated with standard nutritional management, although there may be a need for surgical revision or conversion to RYGB (125).

### 4.7. Kidney Stones

Malabsorptive surgeries are associated with a higher risk of kidney stones after surgery (107). Besides kidney stones, there is a long-term risk of hyperoxaluria and oxalate nephropathy after BS. The risk of new kidney stone events doubles compared with unoperated obese controls (71). Early recognition of these complications with dietary manipulations can successfully reduce oxalate excretion and potential stones. Importantly, the net effect on long-term kidney health and chronic kidney disease risk is often positive after BS when considering the resolution of diabetes and the reduction in pathologic albuminuria (46).

### 4.8. Altered Intestinal Bile Acid Availability

Circulating bile acids increase after surgical procedures (such as RYGB and SG) to raise the blunted levels related to severe obesity. Bile acids, whose functions cover intestinal lipid absorption and various aspects of metabolic regulation, may also play a role in the mechanisms regulating weight loss and glycemic improvements after BS. Bile acids have been shown to have numerous metabolic effects after surgery, such as increasing energy expenditure and gut hormone production, reducing food intake, and improving gluconeogenesis and insulin resistance (104, 144). Overall, the rise in bile acids may explain why fat malabsorption is not as compromised after RYGB surgery and why glycemic control is improved compared to other types of surgeries (i.e., BPD/DS).

### 4.9. Microbiome and Bacterial Overgrowth

The gut microbiome actively plays a role in obesity and conditions such as diabetes and inflammation. For example, the transfer of gut microbiota from obese rodents to germ-free controls leads to an increase in food intake and body weight in nonobese controls (145). Zhang et al. (159) were the first to show a decrease in *Clostridium* bacteria (phylum Firmicutes) after RYGB compared with controls. RYGB surgery reverses the gut microbiota from an obese to a lean phenotype. RYGB-induced changes in the gut microbiota show the standard decrease in the phylum Firmicutes that accompanies weight loss with dieting alone. Microbial sequencing analyses indicate that there is also a decrease in the Bifidobacteriaceae family, and an overabundance of the phyla Proteobacteria and Bacteroidetes, the family Streptococcaceae, and the species *Akkermansia muciniphila* and *Streptococcus salivarius* (27, 33, 82, 97). Importantly, these RYGB findings suggest that gut bacteria correlate with energy intake, high body weight, and inflammation and insulin resistance. Evidence also shows that the RYGB-induced modifications of gut microbiota are associated with changes in white adipose tissue gene expression (64). Hence, a change in the gut microbiota may ameliorate obesity-related symptoms and diseases after RYGB or other bariatric procedures.

The hypochlorhydria and pH-induced increases in the gut after RYGB can be expected to affect the gut microorganisms and may also influence vitamin deficiencies after BS. Because microbial changes after BS vary due to the type of surgery and potential confounders such as baseline diet, medications, and T2DM, the individualized response is less predictable (33).

Probiotic supplementation can alter the microbiota and has been examined to a limited extent after BS. In an early study, Woodard et al. (154) showed that supplementation with *Lactobacillus* probiotics (phylum Firmicutes) compared to placebo led to less breath hydrogen production and bacterial overgrowth and greater weight loss in post-RYGB patients. Others have shown that probiotic supplementation with *Lactobacillus acidophilus* and *Bifidobacterium lactis* Bi-07 was beneficial to markers of the metabolic profile (108). A meta-analysis (150) examining 11 randomized controlled trials (RCTs) indicates that probiotics taken after BS may improve lipid metabolism and liver enzymes and reduce food intake and body weight; severe side effects were not observed (150). Prebiotics have also been evaluated yet have not consistently shown positive effects on the gut microbiota (44, 157). One RCT suggested that a combination of prebiotics (inulin and oligofructose) and probiotics (yogurt) can be beneficial in the early postoperative period (21). A greater understanding of the gut microbiome may explain health outcomes after BS. It may also contribute to defining new treatment modalities in obese patients who elect to undergo BS or those who choose nonsurgical options.

### 4.10. Bone Loss

Bone loss occurs with moderate weight loss (120, 160) and is greater after BS procedures, which is attributed to both the extent of weight loss and other factors such as the type of surgery and extent of malabsorption (76, 158). In addition, vertebral bone strength and density are reduced in adolescents after sleeve gastrectomy (56). A reduction in estrogen, an increase in circulating parathyroid hormone (PTH) and bone resorption, and reduced Ca absorption are some factors contributing to the bone loss (23, 51, 126).

There is a dramatic 33% decrease in Ca absorption at 6 months after RYGB surgery in women consuming 1.5 g/day of Ca and 400 IUs of vitamin D. In a subsequent study, researchers found that even with optimization of vitamin D status, Ca absorption decreased dramatically (117) and the results were similar in patients with SG and RYGB procedures (156). In addition, a prebiotic (soluble corn fiber) was given for 2 months to patients with a history of gastric bypass (~5 years earlier). In this double-blind RCT, the soluble corn fiber did not improve Ca absorption, but those with greater microbial diversity after treatment showed higher Ca absorption (157).

The effects of BS on areal and volumetric bone mineral density (BMD) and microarchitecture occur within the first months. Deterioration progresses over time (26, 45, 158). A meta-analysis of 22 studies with 1,905 patients with obesity who underwent SG found that they had lower BMD at the hip and femoral neck but not at the lumbar spine (59). Thus far, there is no evidence that serum 25-hydroxy-vitamin D [25(OH)D] levels or dietary supplementation of vitamin D or Ca intake modifies the extent of bone loss (45, 51, 126). This is consistent with our early findings of elevated bone resorption and secondary hyperparathyroidism four years after RYGB compared to weight- and age-matched controls, despite a modest intake of calcium and vitamin D (51). One study observed no change in serum PTH or 25(OH)D one year after RYGB surgery, despite doubling dietary calcium and vitamin D intake to approximately 2,350 mg/day and 1,700 IU/day, respectively (45).

**Figure 3:**
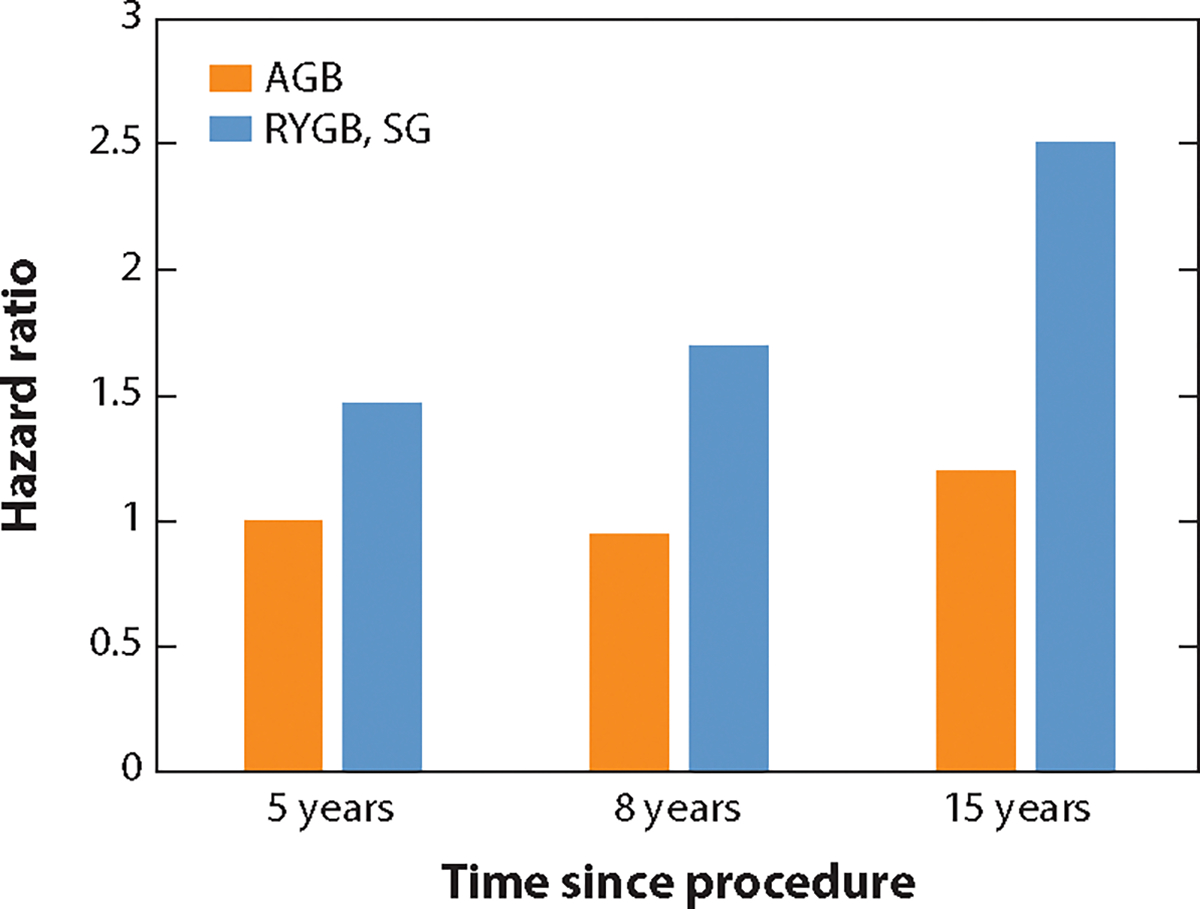
Fracture risk 5–15 years after bariatric procedures. Abbreviations: AGB, adjustable gastric band; RYGB, Roux-en-Y gastric bypass; SG, sleeve gastrectomy.

Fracture risk is increased after BS, but this is generally limited to malabsorptive procedures (3, 72, 96) (Figure 3). Restrictive procedures generally do not increase fracture risk, which can be partially attributed to normal nutrient absorption after these procedures but may also be due to less weight loss compared to malabsorptive procedures or a lack of long-term studies. A meta-analysis of observational studies compared fracture risk between subjects who underwent BS (all types) (*n* = 116,205) and nonsurgical patients (*n* = 134,637) (24). There was a 20% increased risk of any fractures in the BS group compared to the control group (24). Overall, the type of BS, the amount of weight loss, and older age, but not necessarily higher-than-normal serum 25(OH)D levels, influence the extent of bone loss and fracture, as well as indices of bone resorption, serum PTH, and calcium absorption.

## 5. SPECIAL POPULATIONS AND CLINICAL CIRCUMSTANCES

### 5.1. Pregnancy

The weight loss and reduction in adipose tissue resulting from BS in women of reproductive age have been shown to significantly improve fertility (122). However, it is important to consider that the compromised nutrient absorption following BS, combined with increased nutrient and energy demands during pregnancy, can exacerbate deficiencies and affect maternal and fetal growth and development (85). While there are specific nutrient considerations, such as vitamins A, D, C, E, B_9_, and B_12_, zinc, thiamin, iodine, and omega-3s (147), to ensure a normal and safe development of the fetus, there is currently no consensus on adequate supplementation.

Limited evidence suggests that specific vitamin and mineral supplements tailored for pregnancy after RYGB can result in higher blood serum levels of ferritin, hemoglobin, vitamin B_12_, and vitamin D compared to standard pregnancy multivitamins (133). As these customized BS supplements are relatively new in the market, further studies are needed to confirm these findings. It is recommended to wait until 12–18 months after BS, once maximal weight loss and weight stabilization have been achieved, before considering pregnancy. The first year after BS is critical for ensuring adequate nutrient status (75, 93). Ideally, planning for pregnancy should occur prior to BS to ensure not only sufficient nutrient intake and adherence to prescribed supplements but also the adoption of a healthy eating pattern by the woman during pregnancy (47). Supplementation should be tailored and prescribed by a specialist who can provide comprehensive evaluation and follow-up, as both excessive and deficient doses of certain micronutrients can have detrimental effects (147). Nutritional status should be closely monitored before pregnancy, during pregnancy, and throughout lactation to ensure the safety of both the mother and the baby (130, 137).

### 5.2. Adolescents

Well-designed prospective observational studies indicate that weight loss surgery is a safe and effective treatment option for pediatric patients younger than 18 years old, particularly when performed in specialized metabolic and BS centers that have experience working with youth and their families (54). Regular monitoring of dietary adherence and nutritional assessment, as well as education, is crucial for adolescents undergoing surgery due to their changing body composition, growth, and sexual development (40). Recent data revealing the presence of multiple micronutrient deficiencies following metabolic surgery and BS emphasize the importance of ongoing and long-term monitoring (54, 94). It should be noted that malabsorptive procedures are associated with a higher prevalence of nutritional deficiencies after surgery, underscoring the significance of receiving care from specialized centers (111). A comprehensive approach to care, involving shared decision making that is nonstigmatizing and includes the patient and their family, is essential in addressing obesity as a chronic condition throughout the individual’s life span. This approach also necessitates transition planning to adult care for adolescents with obesity, ensuring continuity of care and support (124).

### 5.3. Older Adults

Age is a risk factor for sarcopenic obesity in BS candidates, which is particularly prevalent among women aged 60 years and older (80). Sarcopenia, characterized by significant changes in body composition, can have substantial implications for older adults seeking weight loss surgery. It is associated with physical disability, frailty, diminished quality of life, and increased mortality (31). BS leads to a loss of approximately 8 kg of LBM within the first year after surgery, with a significant proportion of LBM loss occurring in the initial three months. This loss of LBM coincides with a period of reduced energy and protein intake (90). Consequently, the early postoperative period presents an optimal opportunity to implement interventions targeting LBM preservation. Interventions may involve prehabilitation programs for individuals identified with sarcopenia before weight loss surgery, or rehabilitation programs for those who develop sarcopenia during follow-up due to factors such as aging, loss of LBM resulting from weight loss, inadequate protein intake, and insufficient physical activity. These programs should include individualized prescriptions for protein intake and strength training (12).

Various tests and tools are available to assess and characterize sarcopenia in clinical practice and research. Incorporating these assessments into the evaluation of older adults undergoing weight loss surgery is crucial (90). Additionally, it is valuable to include clinical outcomes, such as physical rehabilitation, muscle strength, and muscle function, to better understand the long-term effects of BS on overall health, considering its impact on sarcopenia and related factors (90).

## 6. NUTRITIONAL MANAGEMENT AFTER BARIATRIC SURGERY: THE IMPORTANCE OF AN INTEGRATED PROGRAM

People living with more extreme forms of obesity undergoing BS should be encouraged to participate in a well-structured, specific, and interdisciplinary patient-centered program, including primary care physicians (70, 92). Nutritional complications can be prevented or managed with preoperative assessment and education with high-quality information to enhance patient empowerment and promote self-management (142). Recent qualitative data indicate that adopting person-centered care techniques positively influences patients’ experience with micronutrients and overall nutrition care (70). Dietary management of the common nutritional complications is outlined in Table 2.

### 6.1. Preoperative Management

A preoperative nutritional assessment is crucial as it allows for the identification and correction of any deficiencies (93, 99). Moreover, it provides an opportunity to establish a strong patient-provider relationship. After all types of bariatric procedures, it is recommended to take a complete multivitamin and mineral supplement daily, containing essential nutrients such as thiamin, iron, selenium, zinc, and copper (93, 99). Based on limited evidence, current BS guidelines for SG and RYGB suggest a minimum of 60–80 g/day of dietary protein or 1.0–1.5 g/kg of ideal body weight as an achievable and meaningful target to minimize postsurgical complications (75, 99). Procedures with a higher risk of protein-energy malnutrition may require a protein intake of 90 g/day or up to 2.1 g/kg of ideal body weight. However, due to the limited stomach capacity and increased satiety experienced after BS, achieving these protein targets solely through diet can be challenging. Therefore, dietary protein supplementation has been suggested as a valuable tool to meet daily protein needs (99).

### 6.2. Postoperative Management

To optimize outcomes and prevent complications, access to specialized dietetic support is crucial for individuals with obesity undergoing BS (92). Nutritionists or dietitians will regularly monitor laboratory analyses and individualize and adjust nutritional supplementation as needed (75, 93, 99, 100). Patients should commit to a lifetime of regular monitoring of nutritional intake, dietary and nutritional assessments, identification of obstacles, problem-solving, and ongoing support by either specialized bariatric centers or trained primary care providers (92, 99, 100).

After completion of the program, long-term follow-up should include annual monitoring of nutritional status as part of a shared care model (92, 99). Adherence to bariatric support groups in the long-term has also been shown to be beneficial for weight loss outcomes (5).

As with other chronic conditions, the management of obesity requires an interdisciplinary approach that includes primary care physicians (70, 75, 92, 93, 99, 100). Dietitians or nutritionists play a critical role in assessing and providing ongoing nutrition care for patients who have undergone BS. Their continuous assessment and involvement are integral to ensuring successful long-term bariatric care (92, 100).

### 6.3. Nutrition Support: Enteral and Parenteral Nutrition

Parenteral nutrition (PN) or enteral nutrition (EN) may be necessary to help treat surgical complications from BS and/or malnutrition occurring from a distinct pathology of the gut in patients who have had these procedures (38). BS-related complications can result in malnutrition/failure to thrive with vitamin deficiencies. In a retrospective review, one study identified patients in a home EN database and found that it was typically given by nasogastric tube (149). Home EN was most common after RYGB (74%) compared to other types of BS. Patients were able to meet about 95% of their energy and protein goals. In addition, home PN was primarily administered to patients with RYGB (72%), and the average age of patients in a large database was 52 years old (80% female) (86). Weight increased by 8 kg from the initiation of home PN to the end of treatment, and serum albumin increased from 2.8 to 3.7 g/dL (86).

Both EN and PN can improve nutritional status in the malnourished post-BS patient who is at high nutritional risk and be used as a bridge to better health (38). EN/PN should begin within 3–7 days when it is clear the patient is unable to meet energy needs with normal oral intake. All patients should be given a hypocaloric formula (50–70% of calculated needs) with a moderately high protein intake (1.2 g/kg per day). Home EN/PN can be considered; however, severe malnutrition or the presence of hypoglycemia should prompt hospitalization. Adherence to the clinical practice guidelines (which are routinely updated) by the American or European professional societies is recommended (75).

## 7. CONCLUSIONS

BS is an important tool for weight loss in individuals with severe obesity. It has significant physiological and nutritional consequences that bring a unique mix of new medical and nutritional requirements in this population. Many nutritional deficiencies and complications can be prevented or corrected with careful monitoring and management. Future nonsurgical targets to treat obesity may include a focus on taste/food preferences, gut microbiota, bile acid signaling, and methods to preserve beta cell function and decrease hepatic glucose output, among others. Nonsurgical interventions that mimic the metabolic benefits of BS are a future direction for the obesity field. The glucagon-like peptide-1 (GLP-1) receptor agonists and GLP-1 in combination with glucose-dependent insulinotropic polypeptide (GIP) dual agonists or GLP-1/GIP/glucagon triple receptor agonists are examples of pharmacologic options that can be used with lifestyle changes to target a wider BMI range (60, 61, 153). These pharmacologic options result in a percent weight loss approaching the magnitude of surgical weight loss, but compliance decreases over time, and therefore it is likely that long-term success will involve a combination of techniques (65) using individualized patient care. Monitoring nutritional status to avoid deficiency conditions that are associated with greater and faster weight loss is needed to complement all of these strategies. There is also a need for health-care professionals to be given the resources to address the long-term concerns in these patients, including ongoing support to maintain a healthy body weight throughout the life span. Action must be decisive, people centered, and integrated to increase our chances of successfully preventing and treating severe obesity.

## Figures and Tables

**Table 1:** Nutritional conditions after bariatric surgery and management

Disease/Condition	Symptoms	Management
Beriberi	Wet beriberi: tachycardia, shortness of breath, hypertension Dry and cerebral beriberi: mental confusion, loss of sensation in the limbs, pain or tingling	Thiamin (B_1_) supplementation The dietary recommended intake for thiamin in multivitamins should be adequate
Anemia	Iron, B_12_, and folate deficiency	Additional supplementation may be needed above a daily multivitamin for folate and B_12_; take iron with vitamin C to enhance absorption
Dumping syndrome	Early: postprandial hypoglycemia Late: nausea, diarrhea, loss of consciousness	Early: limit simple carbohydrate intake; consider acarbose or other alpha-glucosidase inhibitors Late: treat for diarrhea (e.g., increase fluids, reduce fats, avoid lactose, consider Lomotil)
Bowel obstruction	Nausea, vomiting, abdominal pain	Surgical intervention
Gastric ulceration	Abdominal pain, bleeding	Proton pump inhibitors, sucralfate therapy, surgical intervention
Bone loss	Reduced bone mineral density and increased fractures	Vitamin D and calcium supplementation (plus weight-bearing physical activity) Consume dietary and supplemental calcium to a total of 1–1.5 g/day (avoid >2 g/day) Treat vitamin D deficiency with standard recommendations

Conditions with the most evidence include the following: Roux-en-Y gastric bypass, adjustable gastric band, vertical sleeve gastrectomy, and biliopancreatic diversion/duodenal switch. Table adapted from Shapses et al. (119) with permission from Springer Nature.

**Table 2:** Dietary management of gastrointestinal symptoms

Symptom	Management
Dysphagia causing coughing or choking	Eat slowly, avoid dry or hard foods (such as crackers or meat) Add more semisoft foods and avoid acidic or spicy foods, as needed
Vomiting	Eat small bites and chew slowly Separate fluids when consuming a meal Eat small, regular meals (every 2–4 h) and rehydrate Monitor electrolytes
Dehydration	Consume 1.5 L/day or more of water, especially after exercise and when exposed to hot weather or conditions Greater fluid intake can also be achieved with adding flavors (lemon, herbs) to water
Constipation	Increase fluids and fiber; consider medications

## SUMMARY POINTS

1. Bariatric surgery is an important weight loss tool in individuals with severe obesity and is currently the most effective long-term weight loss treatment that lowers obesity-related comorbidities.
2. Bariatric surgery also has significant physiological and nutritional consequences that can result in gastrointestinal complications and micronutrient deficiencies.
3. Common clinical events negatively affecting nutritional status include malabsorption, dumping syndrome, kidney stones, altered intestinal bile acid availability, bowel obstruction, ulcers, gastroesophageal reflux, and bacterial overgrowth.
4. Reduced dietary quality and intake, altered nutrient absorption, and inadequate protein intake and vitamin/mineral supplementation are risk factors for poor nutritional status and excess loss of lean body mass and bone.
5. There are unique concerns in adolescents, older individuals, and individuals who become pregnant postoperatively.
6. Successful long-term weight loss will likely require a combination of weight loss techniques and will need to be individualized.
7. With careful management, health-care professionals can minimize the risk of acute and long-term nutrition complications after bariatric surgery.
